# *Lactobacillus acidophilus* protects against *Corynebacterium pseudotuberculosis* infection by regulating the autophagy of macrophages and maintaining gut microbiota homeostasis in C57BL/6 mice

**DOI:** 10.1128/msystems.00484-24

**Published:** 2024-06-27

**Authors:** Dengliang Li, Yuecai Jiang, Zhanding Cui, Mengzhen Ma, Fang Zhu, Guanhua Li, Haoyue Yang, Shaofei Li, Tianliang Zhang, Dekun Chen, Wentao Ma

**Affiliations:** 1 Veterinary Immunology Laboratory, College of Veterinary Medicine, Northwest Agriculture and Forestry University, Yangling, Shaanxi, China; 2 State Key Laboratory for Animal Disease Control and Prevention, College of Veterinary Medicine, Lanzhou University, Lanzhou Veterinary Research Institute, Chinese Academy of Agricultural Sciences, Lanzhou, Gansu, China; 3 Shaanxi Qianyang Saanen dairy goats Development Co., Ltd, Qianyang, Shaanxi, China; University of California San Diego, La Jolla, California, USA

**Keywords:** *Corynebacterium pseudotuberculosis*, *Lactobacillus acidophilus*, macrophages, autophagy, gut microbiota

## Abstract

*Corynebacterium pseudotuberculosis* (*C. p*), a facultative intracellular bacterium, is an important zoonotic pathogen that causes abscesses and pyogenic granulomas. The relationship between gut microbiota and host health or diseases has received increasing attention. However, the role of gut microbiota in the process of *C. p* infection is still unclear. In this study, we established a *C. p* infection model in C57BL/6 mice and examined the impact of preemptive oral administration *Lactobacillus acidophilus* (*L. acidophilus*) on infection. Our findings revealed that *C. p* infection led to pronounced pathological alterations in the liver and kidneys, characterized by abscess formation, intense inflammatory responses, and bacterial overload. Remarkably, these deleterious effects were greatly relieved by oral administration of *L. acidophilus* before infection with *C. p*. Additionally, we further found that during *C. p* infection, peritoneal macrophages (PMs) of mice orally administered with *L. acidophilus* accumulated more rapidly at sites of infection. Furthermore, our results showed that PMs from mice with oral *L. acidophilus* administration showed a stronger *C. p* clearance effect, and this was mediated by high expression of LC3-II protein. Meanwhile, oral administration of *L. acidophilus* protected the gut microbiota disorder in C57BL/6 mice caused by *C. p* infection. In summary, our study demonstrates that oral administration of *L. acidophilus* confers effective protection against *C. p* infection in C57BL/6 mice by modulating macrophage autophagy, thereby augmenting bacterial clearance and preserving gut microbiota and function stability. These findings position *L. acidophilus* as a viable probiotic candidate for the clinical prevention of *C. p* infection.

**IMPORTANCE:**

*Corynebacterium pseudotuberculosis* (C. *p*) is known to induce a range of chronic diseases in both animals and humans. Currently, clinical treatment for C. *p* infection mainly relies on antibiotic therapy or surgical intervention. However, excessive use of antibiotics may increase the risk of drug-resistant strains, and the effectiveness of treatment remains unsatisfactory. Furthermore, surgical procedures do not completely eradicate pathogens and can easily cause environmental pollution. Probiotic interventions are receiving increasing attention for improving the body’s immune system and maintaining health. In this study, we established a C. *p* infection model in C57BL/6 mice to explore the impact of *Lactobacillus* acidophilus during C. *p* infection. Our results showed that *L. acidophilus* effectively protected against C. *p* infection by regulating the autophagy of macrophages and maintaining intestinal microbiota homeostasis. This study may provide a new strategy for the prevention of C. *p* infection.

## INTRODUCTION

*Corynebacterium pseudotuberculosis* (*C. p*) is a facultative intracellular Gram-positive bacterium characterized by the formation of abscesses and pyogranuloma (1). Infection with *C. p* causes various chronic diseases, including caseous lymphadenitis in sheep and goats, ulcerative lymphangitis in horses, mastitis in cows, and necrotizing lymphangitis in humans (2–5). Due to the good tolerance of *C. p* to the external environment and the difficulty in detecting subclinical infections in animals, the invasion of pathogens into animal herds is often overlooked. In addition, pharmacotherapy for clinical patients is not satisfactory and recurrent, which poses difficulties in controlling and eradicating the disease (6). This further caused serious economic losses to livestock production (7). Consequently, there is an urgent need to develop novel preventive and control strategies to curb the onset and progression of diseases caused by *C. p*.

The intricate relationship between gut microbiota and host health or disease states has been increasingly substantiated in recent research (8). Maintaining a stable and healthy gut microbiota is particularly important for enhancing the body’s immunity and resisting pathogen infections. Numerous studies have highlighted the efficacy of probiotics, particularly lactic acid bacteria such as *Lactobacillus acidophilus*, *Lactobacillus casei*, and *Lactobacillus salivarius*, in mitigating the detrimental effects of pathogen infections (9–11). These probiotics achieve this by modulating the balance of gut microbiota and enhancing host immunity (9–11). For instance, Fang Yang et al. discovered that *Bacteroides fragilis* enhances the expression of IFN-γ by regulating the function of long non-coding RNA (lncRNA), effectively resisting pneumonia caused by *Mycobacterium tuberculosis* (12). In addition, it had been demonstrated that metabolites of beneficial gut bacteria reduced the expression of virulence gene pld and protected C57BL/6 mice against *C. p* infection (13).

Macrophages are important innate immune cells that defend against pathogenic microbial infections. Their role is particularly significant in the process of *C. p* infection (14). The clearance of bacteria during pulmonary infection is mainly achieved through the phagocytosis of alveolar macrophages (15). Additionally, autophagy plays an important role in the infection of cells by pathogens. It usually involves targeting cytoplasmic substances to lysosomes for degradation to eliminate intracellular pathogen infections. Macrophages can eliminate pathogens invading cells through the autophagy pathway (16). *L. acidophilus* and *Bacillus clausii* are potent activators of innate immune responses in murine macrophage cell line RAW264.7 cells (17). There is compelling evidence to indicate that probiotic *Bacillus amyloliquefaciens* induces cellular autophagy by upregulating the expression of autophagy genes *Atg5*, *Atg12*, and *Atg16*, thereby enhancing the intracellular clearance of *Escherichia coli* (18). On the contrary, mutations in the key autophagy gene *Atg16* lead to weakened autophagy, resulting in an increase in the number of Salmonella typhimurium in the intestine, mesenteric lymph nodes, liver, and spleen, and aggravated tissue pathology (19). For the induction of autophagy, cytokines are important factors. For example, compelling evidence has shown that IFN-γ, IL-6, and TNF-α can enhance the antibacterial effect of macrophages by inducing the autophagy of these cells (20–22).

The influence of *L. acidophilus* on host peritoneal macrophage (PM) function as well as the detailed changes in gut microbiota after *C. p* infection have not been fully explored. In this study, we investigated the protective effect of *L. acidophilus* on C57BL/6 mice infected with *C. p*. At the same time, the impact of *L. acidophilus* on macrophage function was further explored, as well as the correlation prediction between gut microbiota and macrophage function. Our study provides new insights for the clinical prevention of *C. p* infection by using *L. acidophilus*.

## RESULTS

### Oral *L. acidophilus* administration relieved liver and kidney lesions induced by *C. p* infection

To investigate the impact of *L. acidophilus* on *C. p* infection, first, we assessed the extent of damage infected by *C. p* infection on the host. C57BL/6 mice were subjected to intraperitoneal inoculation of a strain of *C. p* isolated from clinically ill Saanen dairy goats. We found that 15 days after *C. p* infection, it induced the appearance of white nodule lesions in the liver and kidneys (Fig. S1A and B). Histopathological examination revealed significant infiltration of red blood cells and inflammatory cells, along with structural damage in the liver and kidney tissues (Fig. S1C). The histopathological score showed that the *C. p* infection group was significantly higher than the phosphate-buffered saline (PBS) group (Fig. S1D). Moreover, our findings revealed that the liver and kidneys had a significant substantial of live bacteria following *C. p* infection (Fig. S1E). These findings indicated that *C. p* infection caused liver and kidney damage in C57BL6 mice.

**Fig 1:**
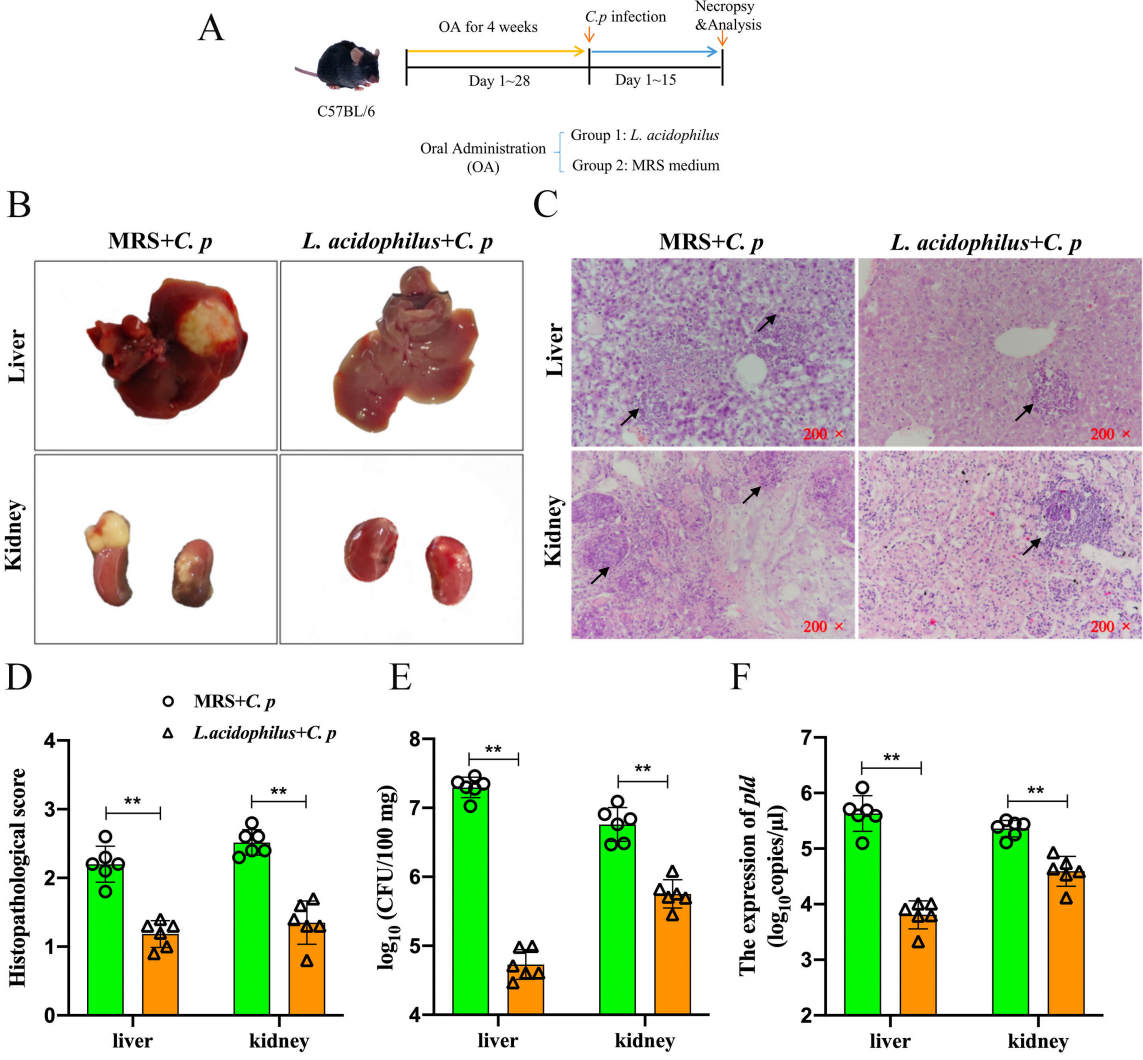
*L. acidophilus* improved the pathological changes induced by *C. p* infection in C57BL/6 mice. (**A**) A schematic illustration of the experimental design. (**B**) The C57BL/6 mice were infected with *C. p* after oral administration of *L. acidophilus* or MRS medium for 4 weeks and then dissected to observe liver and kidney lesions. (**C and D**) Representative images of the hematoxylin and eosin-stained liver and kidneys (original magnification of 200×) and the histopathological score. Black arrows indicated the inflammatory cells and red blood cell infiltration. (**E**) The number of *C. p* from the liver and kidneys at 15 days post infection was determined by the standard plate count method. (**F**) The virulence gene *pld* of *C. p* in the liver and kidneys was detected by quantitative real-time PCR (qRT-PCR) (*P ≤ 0.05 and **P ≤ 0.01; ns represents no significant difference).

Next, we further investigated the role of *L. acidophilus* in the process of *C. p* infection. C57BL/6 mice were administered oral gavage with or without *L. acidophilus* (1 × 10^9^ CFU per day) for 4 weeks before exposure to *C. p* infection (Fig. 1A). The study results indicated that the severity of liver and kidney lesions in mice treated with oral *L. acidophilus* were alleviated (Fig. 1B). Compared to mice treated with De Man-Rogosa-Sharpe (MRS) medium, those given oral *L. acidophilus* exhibited significantly diminished tissue pathology, whereas MRS-treated mice demonstrated more extensive liver and kidney damage characterized by widespread structural deterioration (Fig. 1C and D). Notably, the bacterial load of *C. p* in liver and kidney tissues was significantly lower in the *L. acidophilus*-treated group (Fig. 1E). Furthermore, we established a standard curve to detect the expression of *pld* by using a PGM-T vector plasmid containing virulence genes *pld* (Fig. S2). Consistent with the results of *C. p* burdens, the virulence gene *pld* in liver and kidney was significantly reduced in the *L. acidophilus*-treated mice (Fig. 1F). These findings collectively indicated that *L. acidophilus* acted as a beneficial regulator in relieving liver and kidney pathology induced by *C. p* infection.

### *L. acidophilus* enhanced antimicrobial activity of peritoneal macrophages

**Fig 2:**
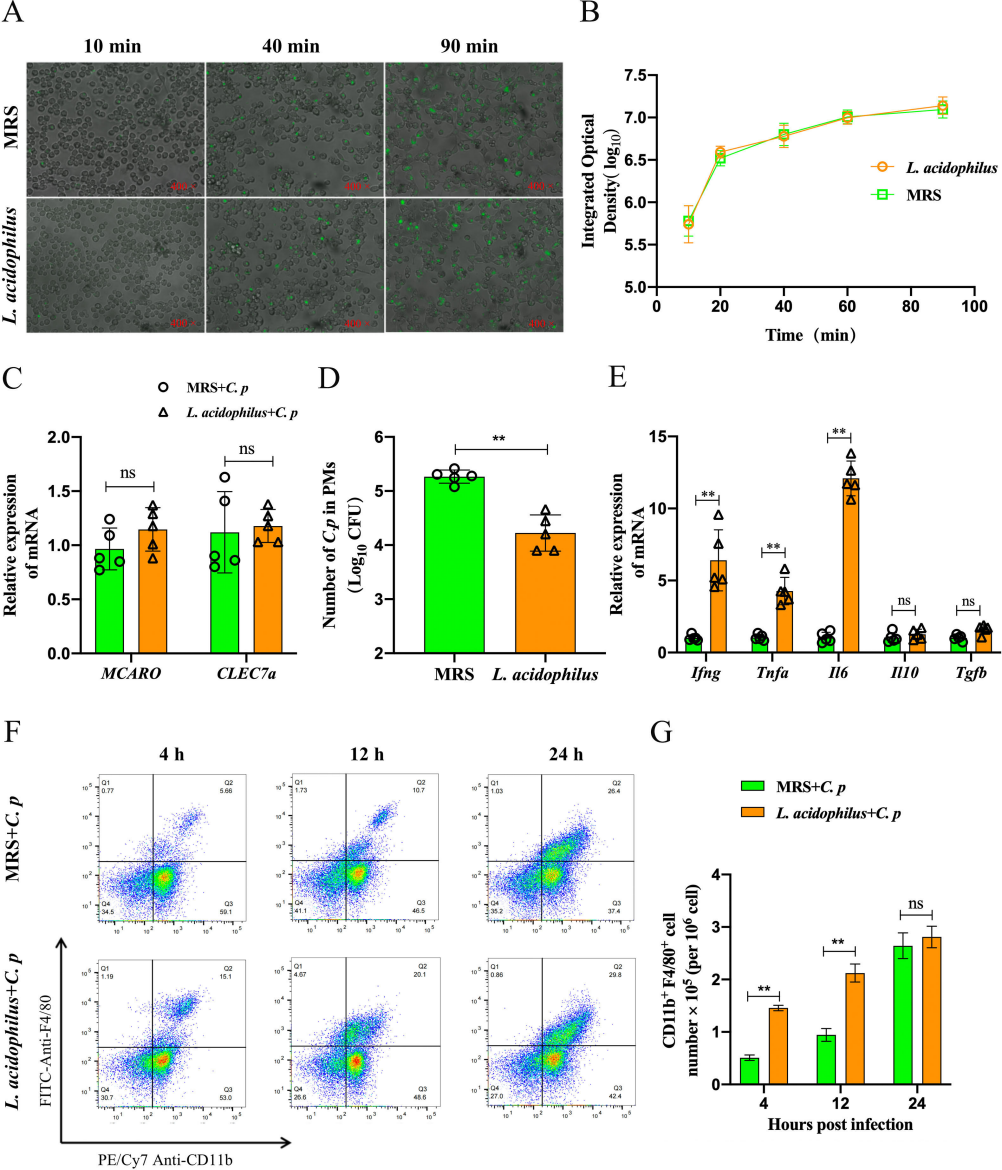
*L. acidophilus* enhances the antimicrobial activity of PMs. (**A–C**) The PMs from C57BL/6 mice with oral *L. acidophilus* or oral MRS medium were collected and infected with *C. p* [multiplicity of infection (MOI) = 10]. (**A**) The phagocytic capacity of PMs was assessed toward FITC-*C. p* through intracellular immunofluorescence counting at different points in time. (**B**) The statistical results of the phagocytic capacity of MPs were expressed by the value of integrated optical density. (**C**) Relative mRNA expression of *MARCO* and *CLEC7A* in PMs after 4 h of infection with *C. p*. (**D**) The PMs were infected for 1 h with *C. p* followed by gentamicin treatment for 6 h before cell lysis. The final result represented the number of viable bacteria within the cell by counting the CFU. (**E–G**) The C57BL/6 mice with oral *L. acidophilus* or MRS were infected with *C. p* (2 × 10^6^ CFU). (**E**) The PMs were collected, and the mRNA expression of *Ifng*, *Tnfa*, *Il6*, *Il0*, and *Tgfb* was detected by qRT-PCR. (**F**) The peritoneal lymphocytes were collected, and the proportion of CD11b^+^ F4/80^+^ cells was detected by flow cytometry. (**G**) The number of CD11b^+^ F4/80^+^ cells was calculated based on the percentage of total lymphocyte counts (*P ≤ 0.05 and **P ≤ 0.01; ns represents no significant difference).

To further elucidate the anti-infective effect of oral *L. acidophilus* during *C. p* infection, we investigated the phagocytic capacity, cytokine expression, and antibacterial activity of PMs in C57BL/6 mice administered with or without *L. acidophilus*. Initially, the phagocytic capacity of PMs was assessed. We found that the phagocytosis of FITC-*C. p* by PMs increased with time, reaching its maximum value at 60 minutes, and there was no significant difference between the oral *L. acidophilus* group and the control group (Fig. 2A and B). Parallel findings were noted in the phagocytosis of enhanced green fluorescent protein (eGFP-labeled) *E. coli* by PMs (Fig. S3). Besides, we detected the mRNA expression of phagocytic receptors *MARCO* and *CLEC7A* in PMs and found no significant difference between the oral *L. acidophilus* group and the control group (Fig. 2C). We also detected the expression of cytokines in PMs and found that PMs of the *L. acidophilus* group showed a significant increase in *Ifng*, *Tnfa*, and *Il6* mRNA expression compared to control PMs after *C. p* infection, while *Il10* and *Tgfb* levels remained similar to controls (Fig. 2E).

To investigate the antibacterial activity of PMs on *C. p* infection, we conducted a gentamicin protection assay. The results showed that the bacterial count in PMs from the *L. acidophilus* group was significantly lower than that in the MRS medium group (Fig. 2D). This indicated that the PMs from the *L. acidophilus* group had a more effective clearance function on *C. p* infection. Additionally, we observed alterations in the quantity of PMs in C57BL/6 mice at various time points following *C. p* infection. The results found that the number of F4/80^+^ CD11b^+^ cells in the abdominal cavity showed an upward trend after infection with *C. p*. And compared with the control group, the *L. acidophilus* group had more F4/80^+^ CD11b^+^ cells at 4 and 12 h after *C. p* infection (Fig. 2F and G). These observations indicated that C57BL/6 mice in the *L. acidophilus* group exhibited faster recruitment of macrophages in the abdominal cavity after *C. p* infection. Taken together, these results indicated that the presence of *L. acidophilus* during the *C. p* infection enhanced PM antimicrobial activity and the mRNA expression of *Ifng*, *Tnfa*, and *Il6* without affecting PM phagocytosis.

### *L. acidophilus* enhanced the elimination of *C. p* in PMs via the autophagic pathway

**Fig 3:**
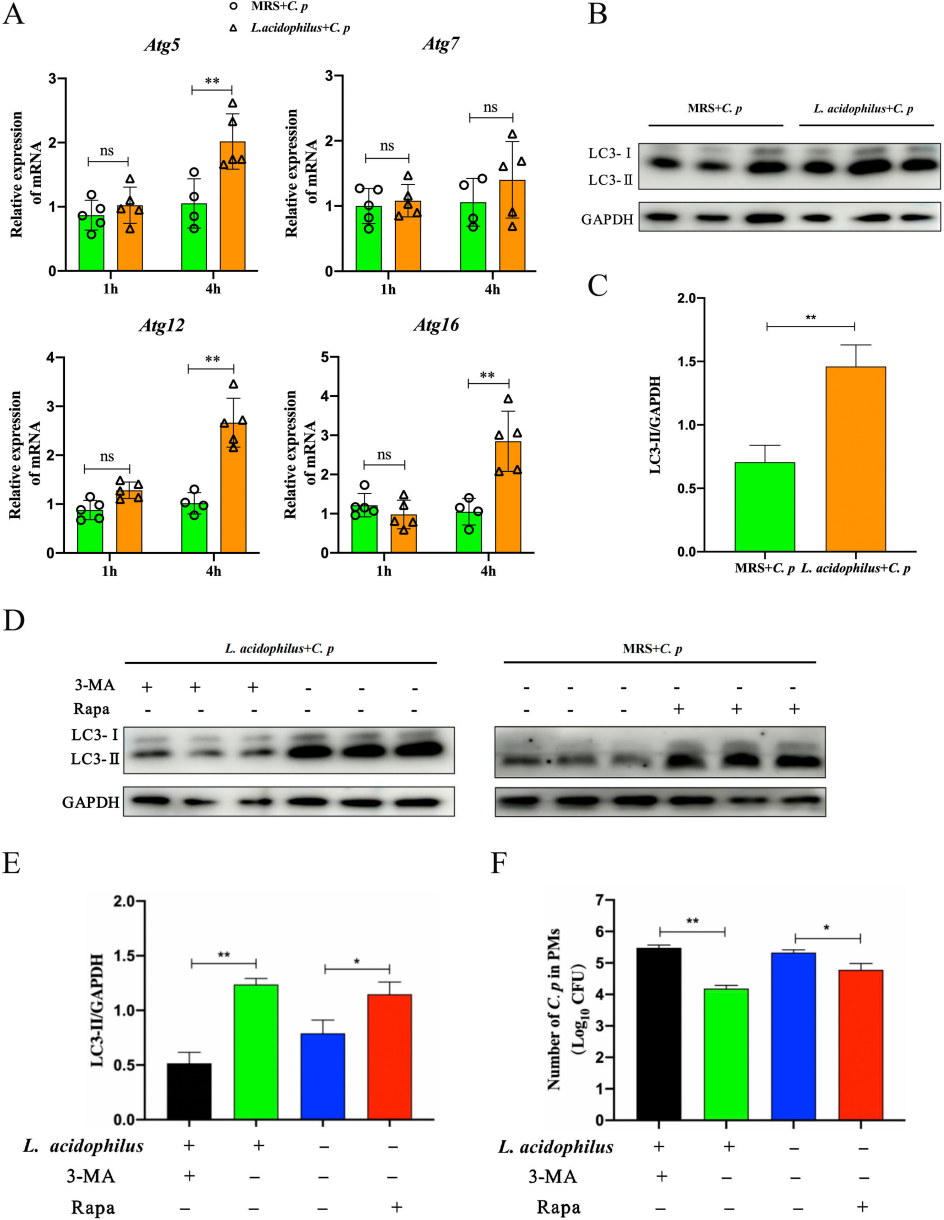
*L. acidophilus* enhanced the elimination of *C. p* in PMs via the autophagic pathway. The PMs from C57BL/6 mice with oral *L. acidophilus* or oral MRS medium were collected and infected with *C. p* (MOI = 10). (**A**) The mRNA expressions of *Atg5*, *Atg7*, *Atg12*, and *Atg16* were detected by qRT-PCR after being infected with *C. p* for 1 h or 4 h. (**B and C**) After 6 h of PMs infection with *C. p*, the LC3 protein expression was determined using western blotting and quantified using Image J software. (**D–F**) The PMs from the oral *L. acidophilus* group were pretreated with 3-MA (2 mM, 3 h), and then infected with *C. p* for 1 h. The PMs from the oral MRS group were pretreated with rapamycin (2 µM, 3 h), and then infected with *C. p* for 1 h. Then PMs were treated with gentamicin for 5 h for subsequent experiments. (**D and E**) The LC3 protein expression was determined using western blotting and quantified using Image J software. (**F**) The number of *C. p* in PMs (lysed using 0.5% Triton X-100) was determined by the standard plate count method (*P ≤ 0.05 and **P ≤ 0.01; ns represents no significant difference).

Previous research has established the critical role of autophagy in the eradication of intracellular bacteria (23). In this study, we investigated whether there was a correlation between the enhanced antibacterial activity and autophagy of PMs. First, we detected the mRNA expression of autophagy core genes *Atg5*, *Atg7*, *Atg12*, and *Atg16*. Our findings revealed that there were no significant alterations in the mRNA expression levels of all the examined genes after 1 h of *C. p* infection (Fig. 3A). The mRNA expression of *Atg5*, *Atg12*, and *Atg16* increased markedly in the PMs of the *L. acidophilus* group after 4 h of *C. p* infection (Fig. 3A). Furthermore, the protein expression of microtubule-associated protein 1 light chain 3 (LC3), an autophagy marker, was examined by western blotting (24). We found that LC3-II was significantly higher in the PMs from the oral *L. acidophilus* group compared with the control group when infected with *C. p* (Fig. 3B and C). These results indicated that the stronger antibacterial activity of PMs in the *L. acidophilus* group might be related to their autophagy. To confirm this hypothesis, we pretreated PMs from the *L. acidophilus* group with autophagy inhibitor 3-MA and subsequently infected them with *C. p*. The expression of LC3-II protein was detected by western blotting, and the number of *C. p* in PMs was tested through gentamicin protection experiments. The results showed that 3-MA significantly inhibited the expression of LC3-II (Fig. 3D and E), and the number of active *C. p* in PMs from the *L. acidophilus* group was significantly higher than that in the untreated group (Fig. 3F). Meanwhile, PMs from the MRS group were remarkably upregulated in LC3-II expression after treatment with the autophagy activator rapamycin, and the number of active *C. p* in PMs was significantly lower than that in the untreated group (Fig. 3D through F). These results indicated that PMs from the *L. acidophilus* group induced stronger expression of autophagy protein LC3-II during *C. p* infection, thereby facilitating the clearance of intracellular bacteria.

### ***L. **acidophilus* modulated the gut microbiota of C57BL/6 mice infected with *C. p*

**Fig 4:**
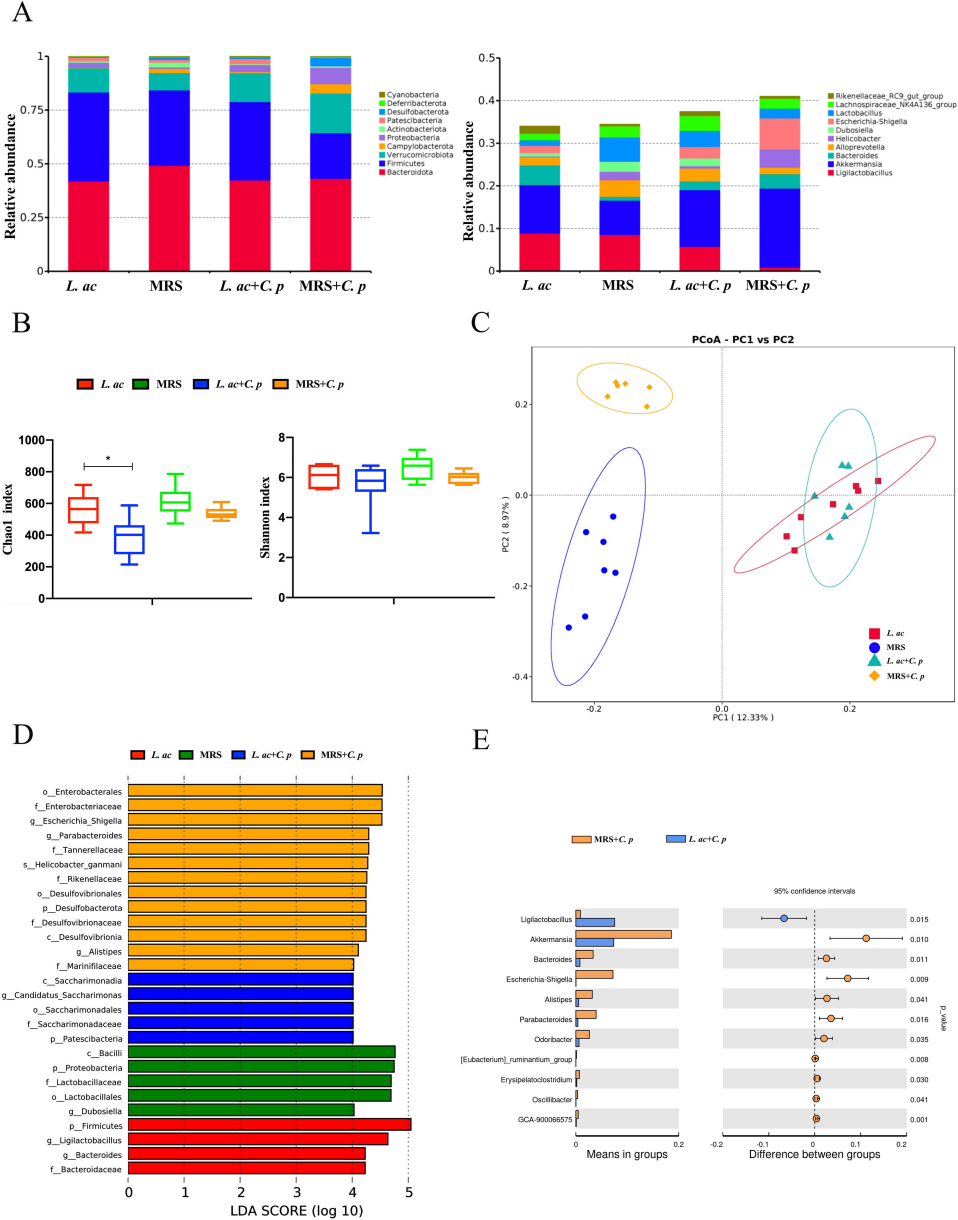
Effects of oral *L. acidophilus* on the gut microbiota of mice infected with *C. p*. The C57BL/6 mice were orally fed with *L. acidophilus* or MRS medium for 4 weeks before collecting fecal samples. Subsequently, the mice were then infected with *C. p*, and fecal samples were collected again 15 days later (*L. ac*: oral *L. acidophilus* without infecting *C. p*; MRS: oral MRS medium without infecting *C. p*; *L. ac + C. p*: oral *L. acidophilus* with infecting *C. p*; MRS + *C. p*: oral MRS medium with infecting *C. p*). (**A**) Average relative abundances of taxa at the phylum and genus level. (**B**) Rarefaction plots based on the chao1 and Shannon indexes. (**C**) Principal component analyses of gut microbiota structure based on weighted UniFrac distance. (**D**) Differentially abundant taxa of gut microbiota among groups, as determined using LEfSe analyses [linear discriminant analysis (LDA) score > 4.0]. (**E**) Comparison of relative abundances of significantly different microbial taxa at the genus level (*P ≤ 0.05 and **P ≤ 0.01; ns represents no significant difference).

To explore the changes in gut microbiota and the effect of *L. acidophilus* on gut microbiota composition during *C. p* infection, we analyzed the gut microbiota of C57BL/6 mice pre- and post-infection with *C. p*. Our results showed that *Bacteroidota*, *Firmicutes*, and *Verrucomimicrobiota* were the dominant phyla in C57BL/6 mice (Fig. 4A). Post infection with *C. p*, there were no significant changes in the relative abundance of bacteria at the phylum level in the *L. acidophilus* group compared with that before infection. However, in the control group treated with MRS medium and infected with *C. p*, a decrease in *Firmicutes* and *Bacteroidota*, along with an increase in *Verrucomimicrobiota* and *Proteobacteria*, was observed. At the genus level, after infection with *C. p* in the control group, *Akkermansia*, *Helicobacter*, and *Escherichia-Shigella* increased, while *Ligilactobacillus* and *Lactobacillus* decreased (Fig. 4A). Moreover, we evaluated microbial diversity within the groups. Indices of bacterial alpha diversity, such as Chao1 and Shannon, indicated higher diversity in the *L. acidophilus* group compared to controls (Fig. 4B). Principal coordinate analyses (PCoAs) indicated that bacterial composition (beta diversity) separated distinctly in groups of control compared to the group with *L. acidophilus* after C57BL/6 mice were infected with *C. p* (Fig. 4C). The results suggested that infection with *C. p* altered the composition of the gut microbiota, whereas pretreatment with *L. acidophilus* helped maintain its stability. Further taxonomic analysis revealed that, prior to infection, the abundance of *Firmicutes*, *Ligilactobacillus*, *Lactobacillaceae*, and *Lactobacillales* was high in both the *L. acidophilus* group and the control group (Fig. 4D). After *C. p* infection, the abundance of some conditional pathogenic bacteria *Escherichia_Shigella* and *Helicobacter_ganmani* increased in the control group (Fig. 4D). While *Saccharimonadia*, *Candidatus_Saccharimonas* in the *L. acidophilus* group increased, which played an important role in maintaining intestinal homeostasis and activating immunity (25). At the genus level, the results showed that *Ligilactobacillus* significantly decreased, while *Akkermansia* and *Escherichia_Shigella* significantly increased after *C. p* infection in the control group (Fig. 4E). These results suggested that the gut microbiota composition of C57BL/6 mice infected with *C. p* was disrupted, while the regulation of *L. acidophilus* maintained the stability of the gut microbiota.

### Prediction and correlation analysis of gut microbiota function

**Fig 5:**
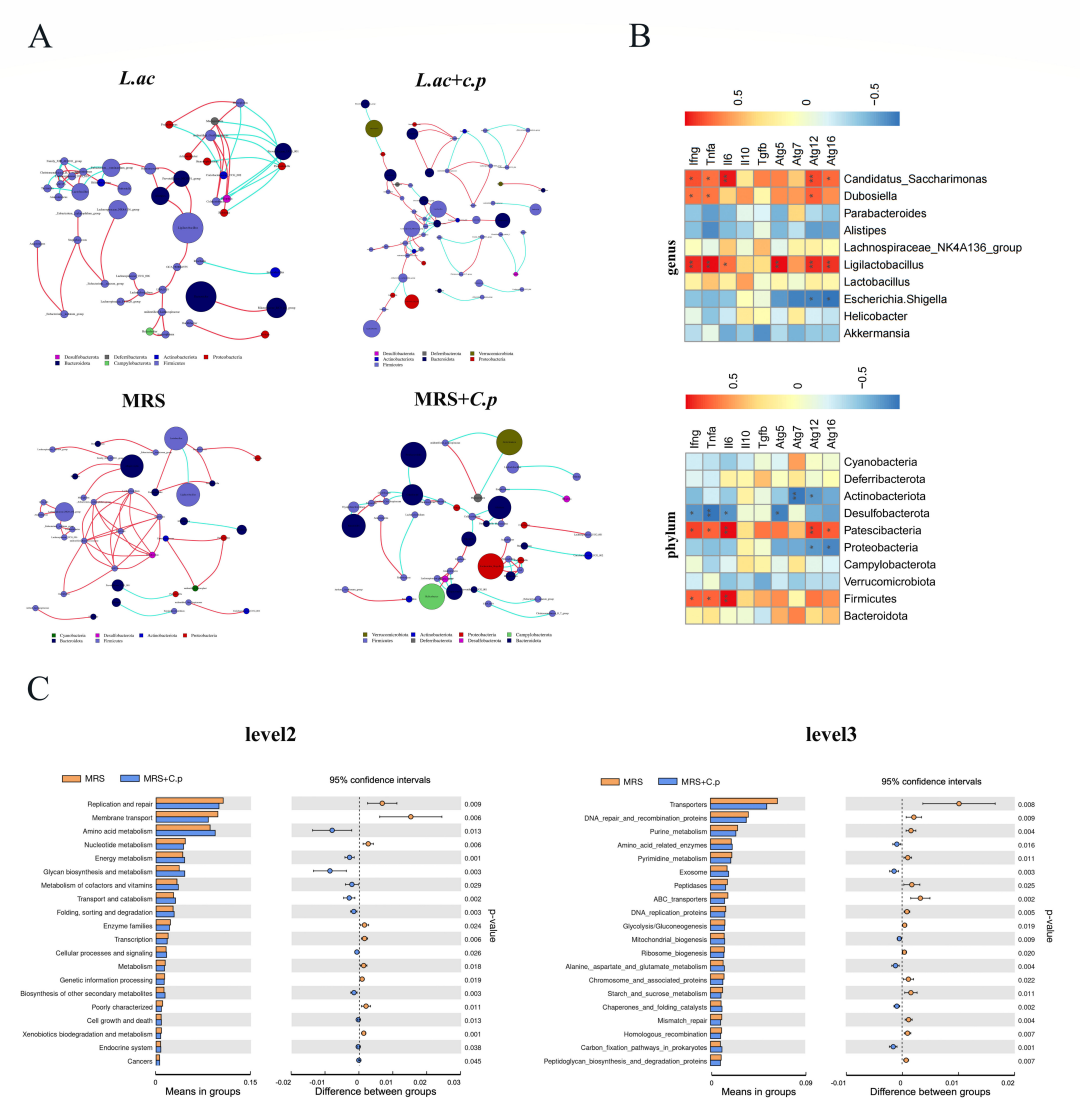
Prediction and correlation analysis of gut microbiota function. (**A**) Analysis of species co-occurrence network at the genus level of the four groups of different bacterial communities for *L. ac*, *L. ac + C. p*, MRS, and MRS + *C. p*. The complex nature of intermicrobial interactions in the ecological community of each group was characterized by co-occurrence networks using graphs. Main groups of co-occurrence species are presented in different colors. Each node in the network indicates a species. The size of each node is proportional to the relative abundance of each species. The red line indicates a positive correlation, and the blue line indicates a negative correlation (remove connections with correlation values <0.6). (**B**) Spearman correlation analysis of microbial diversity and macrophage gene expression. The color bar shows correlation values, where red color indicates a positive association, and the blue color indicates a negative association. (**C**) The Tax4Fun analysis based on the Kyoto Encyclopedia of Genes and Genomes (KEGG) database to predict microbial metabolic function and analyze the functional differences. *t* Test analysis for top 20 metabolic pathway sub-functions of the KEGG signaling pathway at levels 2 and 3 (*P ≤ 0.05 and **P ≤ 0.01).

The interaction of the gut microbiota is very important for the stability of healthy biological communities. Therefore, we explored the bacterial community co-occurrence network of C57BL/6 mice before and after *C. p* infection. The results showed that the oral administration of *L. acidophilus* resulted in slight changes in the microbial community structure of mice before and after *C. p* infection but still retained the main characteristics of the gut microbiota network. The *Firmicutes* remained the dominant phylum and closely interacting species group, including *Ligilactobacillus*, *Lachnospiraceae NK4A136_group*, and *Lactobacillus* (Fig. 5A). However, in the control group treated with MRS medium, there was a significant change in the microbial community structure after *C. p* infection. The dominant phyla of interaction had changed from *Firmicutes* to *Bacteroidetes* and *Proteobacteria* (Fig. 5A). These results indicated that *C. p* infection disrupted the original gut microbiota network interaction structure, while oral administration of *L. acidophilus* maintained the homeostasis of the microbiota network interaction structure.

To explore the relationship between gut microbiota and macrophage function, we conducted a Spearman correlation analysis on gut microbiota and macrophage function indicators. The study found significant correlations between gut microbiota and macrophage function. Among them, *Firmicutes*, *Patescibacteria*, *Ligilactobacillus*, *Dubosella*, and *Candidatus Saccharimonas* were positively correlated with the expression of inflammatory cytokines (*Ifng*, *Tnfa*, and *Il6*) and autophagy genes (*Atg5*, *Atg7*, *Atg12*, and *Atg16*) in macrophages. On the contrary, *Proteobacteria*, *Desulfobacteriota*, *Actinobacteriota*, and *Escherichia-Shigella* were significantly negatively correlated with the indicators of macrophages (Fig. 5B). Kyoto Encyclopedia of Genes and Genomes (KEGG) functional annotation prediction analysis was performed on the gut microbiota using Tax4Fun. The analysis revealed a total of six metabolic pathways were annotated at level 1 (Fig. S4A). *t* Test analysis found significant differences only between the MRS group and the MRS + *C. p* group (Fig. S4B). The 45 KEGG pathways and 387 KEGG pathways were annotated at levels 2 and 3, respectively (not shown). Similar results showed significant differences in *t* test analysis only between the MRS and the MRS + *C. p* group (Fig. 5C). These results indicated that *C. p* infection disrupted the gut microbiota, which had a significant impact on metabolic signaling pathways.

## DISCUSSION

*C. p* is a facultative intracellular bacterium known to induce a range of chronic diseases in both animals and humans. Especially for small ruminant animals, the epidemiological distribution of *C. p* is significant and widely exists worldwide, causing huge economic losses to the breeding industry (7). Currently, there is still no safe and effective prevention and control measure for *C. p* infection (6, 26). In recent years, the role of gut microbiota has become increasingly well-known. The intestinal microbiota is involved in the body’s metabolic function, immune system improvement, anti-infection effects, development, and behavior (27, 28). Previous studies have shown that probiotics can effectively enhance the body’s anti-infective effect against pathogens (29, 30). In this study, we found that preemptive oral administration of *L. acidophilus* effectively prevented *C. p* infection, which was reflected in reducing organ pathology, tissue damage, and bacterial load in the liver and kidneys. Phospholipase D (PLD) is a highly efficient exotoxin and key virulence factor secreted by *C. p* (26, 31). PLD promotes the dissociation of sphingolipids and increases vascular permeability, thereby promoting the infection and transmission of *C. p* (26). We also found that the expression of *pld* genes in the liver and kidneys of the protective group mice was significantly reduced, consistent with the view of Zhou et al. (13), which helps to inhibit the spread of *C. p*.

Probiotics can exert host anti-infection effects by regulating the function of immune cells (9, 32, 33). In this study, we investigated the effect of *L. acidophilus* on macrophage function. Our study found that there was no significant difference in the phagocytic ability of PMs for FITC-*C. p* and eGFP-*E. coli* between the *L. acidophilus* group and the control group. Contrary to our research findings, Deepti Kaushal et al. demonstrated that *L. acidophilus* and *Bifidobacterium bifidum* enhance the phagocytic activity of peritoneal macrophages in elderly mice (34). This may be due to differences between different probiotic strains or animals in different physiological stages. IFN-γ is a key cytokine in response to viral or intracellular bacterial infections (35). Studies have shown that IFN-γ enhances macrophage clearance of intracellular microorganisms through the regulation of signal sensor JAK and transcription activator STAT (36, 37). During *C. p* infection, the expression of TNF-α and IFN-γ helps the body resist infection (38). Consistent with these findings, in our study, the mRNA expression of *Il6*, *Ifng*, and *Tnfa* was significantly increased in PMs with stronger *C. p* elimination effects. In addition, the number of PMs in the *L. acidophilus* group was remarkably higher than that in the control group at 4 and 12 h after *C. p* infection. These findings align with observations in the gut microbiota’s innate immunity against enterovirus systemic infection, underscoring the capacity of certain beneficial gut bacteria to modulate the host’s innate immune system and respond aptly to pathogenic invasions (39).

For macrophages, the common view is that autophagy serves macrophages by enclosing invasive pathogens in autophagosomes and transferring them to lysosomes, where the infection of foreign pathogenic microorganisms is eliminated (40). Previous studies have shown that autophagy-related genes *Atg5*, *Atg12*, and *Atg16* play a crucial role in *M. tuberculosis* infection (41, 42). The deletion of *Atg7* and *Atg14* genes cannot inhibit the replication of *M. tuberculosis* in macrophages (43). Based on the results, our study evaluated the mRNA relative expression of autophagy-related genes in PMs. Interestingly, we found that *Atg5*, *Atg12*, and *Atg16* were more significantly expressed in PMs from *L. acidophilus* pretreatment mice. At the protein level, we also found that the PMs of mice pretreated with *L. acidophilus* exhibited significantly higher levels of autophagy protein LC3-II after infection. This indicated that the stronger clearance effect in PMs for *C. p* might be related to their autophagy. Furthermore, using the autophagy inhibitor 3-MA to block autophagy dramatically reduced the bactericidal activity of PMs from *L. acidophilus* pretreatment mice. This further confirms this viewpoint. Consistent with our findings, other researchers have reported that enhanced autophagy plays an important role in pathogen elimination (44). Although our research has demonstrated that *L. acidophilus* can effectively eliminate *C. p* infection by regulating macrophage autophagy, further research was still needed to fully elucidate the potential molecular and cellular mechanisms.

Our results confirmed that the infection of *C. p* disrupted the original gut microbiota homeostasis, which was crucial for the disease and health of the host (45). The destruction of the established intestinal community structure alters the overall balance between the microbiota and the host, leading to a change in susceptibility to infection (46–48). Consistent with our findings, other investigators have reported that *Toxoplasma gondii*, influenza A virus, *Clostridium difficile*, etc. caused disruption of the gut microbiota and led to more severe intestinal inflammation and diarrhea after infection (49–52). It was worth noting that in our investigation, preemptive oral administration of *L. acidophilus* maintained the homeostasis of the gut microbiota in mice infected with *C. p*. Among them, *Firmicutes*, *Patescibacteria*, and *Ligilactobacillus* were still dominant species after *C. p* infection, which played an important role in maintaining the stability of microbial community structure and function, as well as host resistance to infection (53, 54). Further functional predictions also indicated that the disruption of gut microbiota caused by *C. p* infection can lead to significant changes in metabolic signaling pathways, which might be intricately connected to the host’s systemic immune system and overall health condition (55). Overall, our research has demonstrated that *L. acidophilus* enhanced the antibacterial activity of the host by regulating the function of macrophages. This included rapid recruitment, activation, and enhanced autophagy of macrophages in the early stages of infection. Timely and effective removal of pathogens contributed to maintain homeostasis of gut microbiota structure (45, 46). Correspondingly, a healthy and stable gut microbiota played an important role in maintaining health and combating infections (53, 54). They formed a virtuous cycle of enhancing host immunity and resisting infection. Admittedly, our current research has not yet fully elucidated the exact mechanisms through which the gut microbiota modulates host macrophage function and enhances antimicrobial activity, specifically the molecular pathways involved. So, further in-depth research is needed to clarify the more precise mechanisms of gut microbiota, immune function, and host resistance to infection.

**Fig 6:**
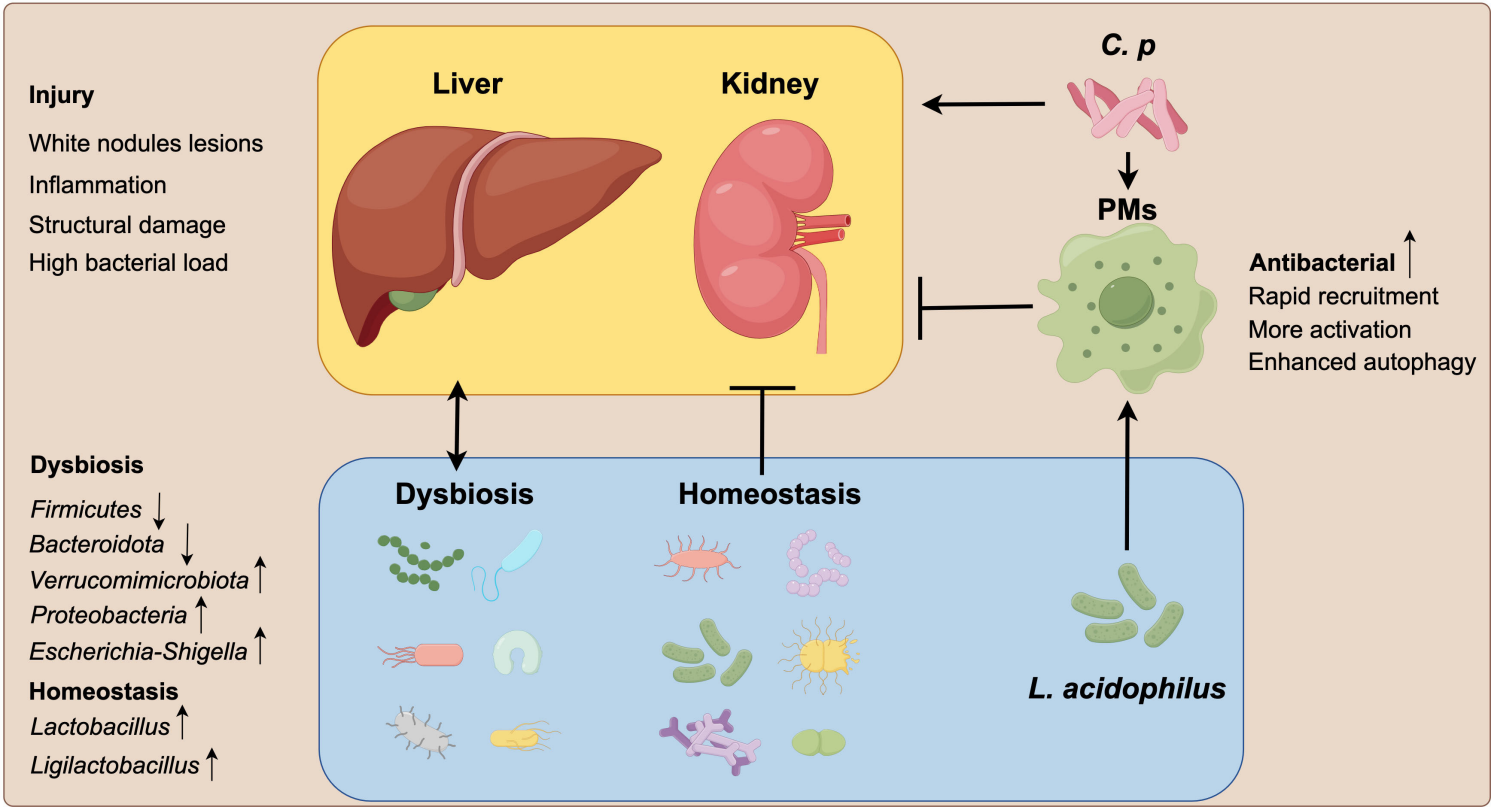
The effect of *L. acidophilus* on the function of host PMs and gut microbiota. The PMs in the *L. acidophilus* group exhibited a more active state during *C. p* infection, characterized by more rapid recruitment, activation, and enhanced autophagy. These enhanced responses resulted in a more effective antibacterial activity, alleviating the tissue and organ damage caused by *C. p* infection and maintaining homeostasis of gut microbiota structure.

In conclusion, oral administration of *L. acidophilus* can effectively protect C57BL/6 mice against *C. p* infection by regulating the autophagy of PMs to enhance the clearance of *C. p* and maintaining a more stable gut microbiota structure (Fig. 6). These findings position *L. acidophilus* as a promising probiotic candidate for the clinical prevention of *C. p* infection, highlighting its potential role in modulating host immunity and gut microbiota homeostasis. Future studies should aim to characterize the molecular dialog between *L. acidophilus* and host cells, particularly focusing on the signaling pathways that lead to the modulation of autophagy and the subsequent enhancement of pathogen clearance. Understanding these mechanisms could unlock targeted strategies to bolster host defenses against *C. p* and other intracellular pathogens.

## MATERIALS AND METHODS

### Bacterial strains and culture conditions

*C. p* was isolated from clinically ill Saanen dairy goats and preserved in our laboratory. The *C. p* was cultured in LB broth supplemented with 0.05% Tween 80 at 37℃ for 48 h. The eGFP-*E. coli* was kindly gifted by Dr. Zhanding Cui from the Chinese Academy of Agricultural Sciences, China. The FITC-*C. p* was constructed and saved by our laboratory. *L. acidophilus* was isolated from fecal samples of healthy Saanen dairy goats and stored in our laboratory. Under anaerobic conditions, *L. acidophilus* was cultured in MRS broth medium at 37℃ for 24 h. Before the oral experiment in mice, the quantification of *L. acidophilus* was performed by plate counting. Briefly, the bacteria were diluted and coated on MRS solid medium plates for cultivation, and the number of viable bacteria was calculated by CFU.

### Animals and treatments

Specific pathogen-free C57BL/6 mice with 6- or 10-week-old were purchased from the Model Animal Research Center of Northwest A&F University. Mice freely obtained food and water in an environment with controllable temperature (25℃ ± 1℃) and relative humidity (50% ± 5%). For the *C. p* infection experiment, 10-week-old C57BL/6 mice (six males and six females) were randomly divided into two groups. The experimental group was infected by intraperitoneal injection of 200 µL PBS containing *C. p* (10^7^ CFU/mL). The control group mice were intraperitoneally injected with an equal dose of PBS. Animals were continuously monitored for 15 days, followed by autopsy analysis (Fig. 1A). The effect of oral administration of *L. acidophilus* on *C. p*-infected mice was studied. Six-week-old C57BL/6 mice (six males and six females) were randomly divided into two groups. The mice of the experimental group were orally administered 200 µL of *L. acidophilus* (1 × 10^9^ CFU) daily for 4 consecutive weeks, and the control group mice were orally treated with MRS medium, followed by *C. p* infection (in the same manner as above). Feces, intestinal contents, liver, kidney, and PMs were collected from mice at designated time points for subsequent experimental analysis.

### Bacterial load detection of liver and kidney

The organs of the liver and kidneys were collected and weighed under sterile conditions. Subsequently, the organizations were homogenized using a tissue grinder and continuously diluted in a 10-fold gradient in PBS. The PBS diluent was coated onto LB solid medium plates and incubated at 37°C for 48 h. The CFU of *C. p* was counted, and the concentration per milligram of organizations was determined.

### Hematoxylin and eosin staining

Liver and kidney tissues were collected and fixed in 4% paraformaldehyde for 72 h. The tissue was embedded in paraffin and then cut into 5 µm slices. After hematoxylin and eosin staining, tissue slices were observed and analyzed under an optical microscope. Refer to previous research for histopathological scoring (56). Briefly, the histopathological scoring was performed on a scale of 0–3: no inflammatory cell infiltration, with basic intact tissue structure (0 score), mild infiltration of inflammatory cells and slight changes in tissue structure (1 score), moderate infiltration of inflammatory cells and moderate changes in tissue structure (2 score), inflammatory cells infiltrate largely and extensively, with severe changes in tissue structure (3 score). Three high-resolution fields (×400) for each slice were observed and scored. Finally, the average value of the three fields was regarded as the final score for the slice.

### Collection of peritoneal macrophages

The mice were euthanized by cervical dislocation and soaked in 75% ethanol for 5 minutes for sterilization. In a sterile workbench, the mouse skin was cut from the abdomen (without opening the peritoneum). The abdominal endothelium was lifted with forceps, and 10 mL of sterile PBS (0.1 M) was injected into the abdominal cavity using a 5 mL syringe. The peritoneum was pressed for 5 minutes, the abdominal fluid was sucked back into the syringe and transferred into a centrifuge tube. The collected peritoneal fluid was centrifuged at 800 g for 5 minutes, and the supernatant was discarded. The cells were resuspended in RPMI 1640 (Sigma-Aldrich) with 10% fetal bovine serum (Gibco) and counted. Next, the cells were transferred to a six-well cell plate and cultured at 37℃ with 5% CO_2_. After 4 h, the non-adherent cells were washed with PBS, and the adherent cells were digested with 0.05% trypsin (Sigma-Aldrich) to obtain PMs (14).

### Phagocytosis assay

The phagocytic activity of PMs was detected by fluorescently labeled bacteria. The PMs were infected by FITC-*C. p* (or eGFP-*E. coli*) with 10 MOI and incubated at 37°C and 5% CO_2_ for the indicated time (15 min, 20 min, 40 min, 60 min, and 90 min). The cells were washed with PBS, and a 4% trypan blue solution was added to the cell culture well to quench the fluorescent bacteria adhering to the outside of the cells. Cell fluorescence was observed through a fluorescence microscope. The integrated optical density was analyzed through Image-Pro Plus software (Roper Technologies, Sarasota, Florida, USA) to detect the phagocytic effect of PMs on bacteria.

### Gentamicin protection assay

For the gentamicin protection assay, PMs were infected for 1 h with 10 MOI *C. p*, followed by gentamicin (to a final concentration of 100 µg/mL) treatment for 6 h. Cells were then lysed in 1% TritonX-100 buffer (Sigma-Aldrich) for 40 min, and the lysate was plated on agar plates. The final result represented the number of viable bacteria within the cell by counting the CFU of *C. p*.

### Flow cytometry

Flow cytometry was used to characterize and phenotype activation indicators of PMs. Cells were harvested, washed, and counted. Then PE/Cy7 anti-mouse CD11b (BioLegend) and FITC anti-mouse F4/80 (BioLegend) were used for cell surface staining after blocking with anti-mouse CD16/32 (BioLegend). The PMs were identified as CD11b^+^ F4/80^+^ cells. Samples were analyzed using a BD FACSAria III Flow Cytometer. Subsequent analysis was performed with Flow Jo software (Tree Star Inc., San Carlos, CA).

### Quantitative real-time PCR

We extracted total RNAs from cells and tissues using TRIzol Reagent (Takara). Then, reverse transcription was conducted with a PrimeScript RT Reagent Kit (Takara). We performed qRT-PCR using the QuantStudio 6 Flex Real-Time PCR System (Life Technologies, Carlsbad, CA, USA) with the SYBR Premix Ex Taq II Kit (Takara) to examine the gene expression of samples at the mRNA level by the 2^−ΔΔCt^ method [relative to glyceraldehyde-3-phosphate dehydrogenase (GAPDH) expression]. All primers used in real-time PCR were designed according to the PrimerBank database and Primer BLAST of NCBI. The detailed primer sequences are listed in Table S1.

### Western blot assay

Cells were lysed by RIPA buffer (Beyotime) with 1% protease inhibitors and 1% phosphatase inhibitors (Thermo Scientific), and the protein extract was quantified by a BCA kit (Thermo Scientific). An equivalent amount of protein extract (20 mg) was run on SDS-PAGE gel (12%; Bio Rad) and then transferred to the polyvinylidene difluoride (PVDF) membrane. The membranes were blocked with 5% bovine serum albumin (BioFroxx) for 1 h and then incubated with primary antibodies against LC3 (Cell Signaling Technology) and GAPDH (Thermo Scientific) at 4°C overnight. Then, the PVDF membranes were washed with Tris buffer plus Tween and incubated with second antibodies labeled with horseradish peroxidase (HRP) (anti-mouse IgG or anti-rabbit IgG, Bioss) at room temperature for 2h. After washing three times, we dropped the enhanced ELC chemiluminescence reagent (Yeasen Biotechnology) onto the blots and exposed it using the Amersham Image Quant 800 system (Cytiva Sweden AB). Band intensities were quantified with Image J (version 1.52 a).

### Gut microbiota analyses

The gut microbiota was analyzed through 16S rRNA gene sequencing. Briefly, the total DNA of the gut microbiota was extracted from fecal samples with the QIAamp Fast DNA stool extraction kit (Qiagen, Dusseldorf, Germany). Amplification and purification of genes in the V3–V4 region of bacteria 16S rRNA, followed by library construction and quantitative analysis. Quantified libraries were pooled and sequenced on Illumina platforms according to the effective library concentration and data amount required. Raw data were processed by filtering and removing chimeric sequences using Fastp software to obtain the final effective data. Besides, denoise was performed with DADA2 in the QIIME2 software (Version QIIME2-202202) to obtain ASVs (Amplicon Sequence Variants). Species annotation was performed using QIIME2 software. Alpha diversity was evaluated by Chao1 and Shannon index using QIIME2. In order to evaluate the complexity of community composition and compare the differences between samples (groups), PCoA was performed in QIME2 to obtain principal coordinates from complex and multidimensional data and visualize them. The LEfSe method was used to determine statistically different bacteria among groups (LDA ≥ 4). At the same time, *t* tests (*P* < 0.05) were conducted on the differences in bacteria between groups at the genus level. A model prediction of the correlation network diagram at the genus level was created by calculating the correlation index of all samples. The species correlation coefficient matrix was obtained, and the filtering conditions were set as follows: (i) removed connections with correlation coefficients <0.6, (ii) filtered out node self connections, (iii) removed connections with node abundance less than 0.005% and then obtained the network graph. Furthermore, the metabolic function was predicted by Tax4Fun analysis through the KEGG database. The analysis of gut microbiota was performed with a data analysis platform (https://magic.novogene.com/).

### Statistical analysis

All data in the experimental results were represented as mean ± SE. Statistical comparisons between different groups were performed using an ordinary two-way analysis of variance (ANOVA) followed by post Sidak’s multiple-comparison test or by an unpaired two-tailed Student’s *t* test for two groups using GraphPad Prism (GraphPad Software). When the *P*-value was less than 0.05, it was considered statistically significant (*P ≤ 0.05 and **P ≤ 0.01; ns represents no significant difference). Draw pattern diagrams through the drawing platform software (https://www.home-for-researchers.com/#/).

## Data Availability

All data are available in the main text or the supplemental material.

## SUPPLEMENTAL MATERIAL

Supplemental material msystems.00484-24-s0001.docx Fig. S1 to S4; Table S1.

ASM does not own the copyrights to Supplemental Material that may be linked to, or accessed through, an article. The authors have granted ASM a non-exclusive, world-wide license to publish the Supplemental Material files. Please contact the corresponding author directly for reuse.
