# Supplementary material for: Lactobacillus acidophilus protects against Corynebacterium pseudotuberculosis infection by regulating the autophagy of macrophages and maintaining gut microbiota homeostasis in C57BL/6 mice
Source: mSystems. 2024 Jun 27;9(7):e00484-24. doi: 10.1128/msystems.00484-24 (PMC11265446; doi:10.1128/msystems.00484-24)
Supplement: Supplemental material — Fig. S1 to S4; Table S1. [file msystems.00484-24-s0001.docx]

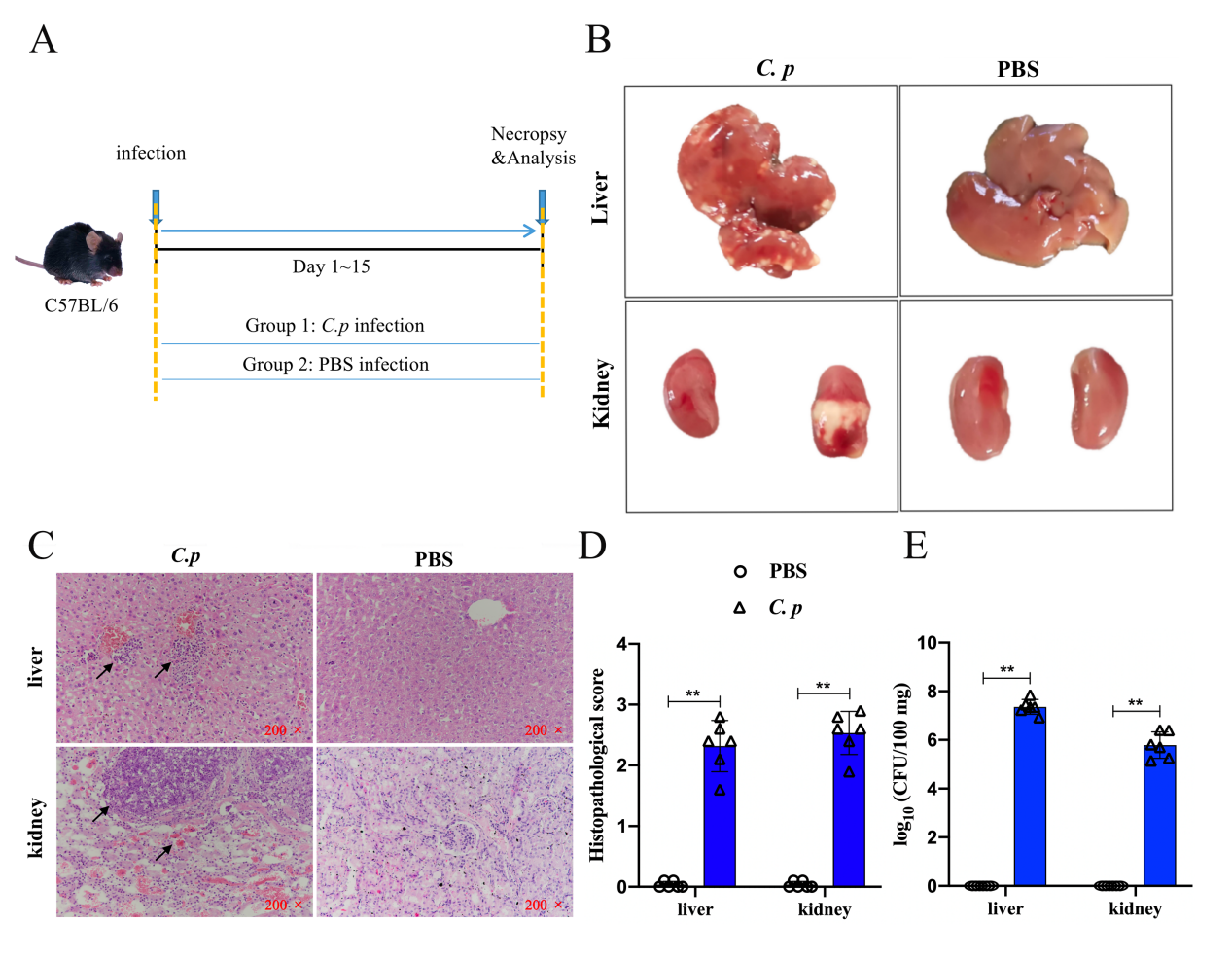


**Fig.S1 Pathological changes induced by *C. p* infection in C57BL/6 mice.** **(A)** Schematic illustration of the experimental design. **(B)** After 15 days of infection with *C. p* or PBS, the C57BL/6 mice were necropsied to observe the gross lesions in the liver and kidney. **(C-D)** Representative images of H&E-stained liver and kidneys (original magnification of 200×) and the histopathological score. Black arrows indicated the inflammatory cells and red blood cells infiltration. (**E**) The number of *C. p* from liver and kidneys at 15 days post infection was determined by the standard plate count method. (* *P* ≤ 0.05, ** *P* ≤ 0.01, ns represents no significant difference)


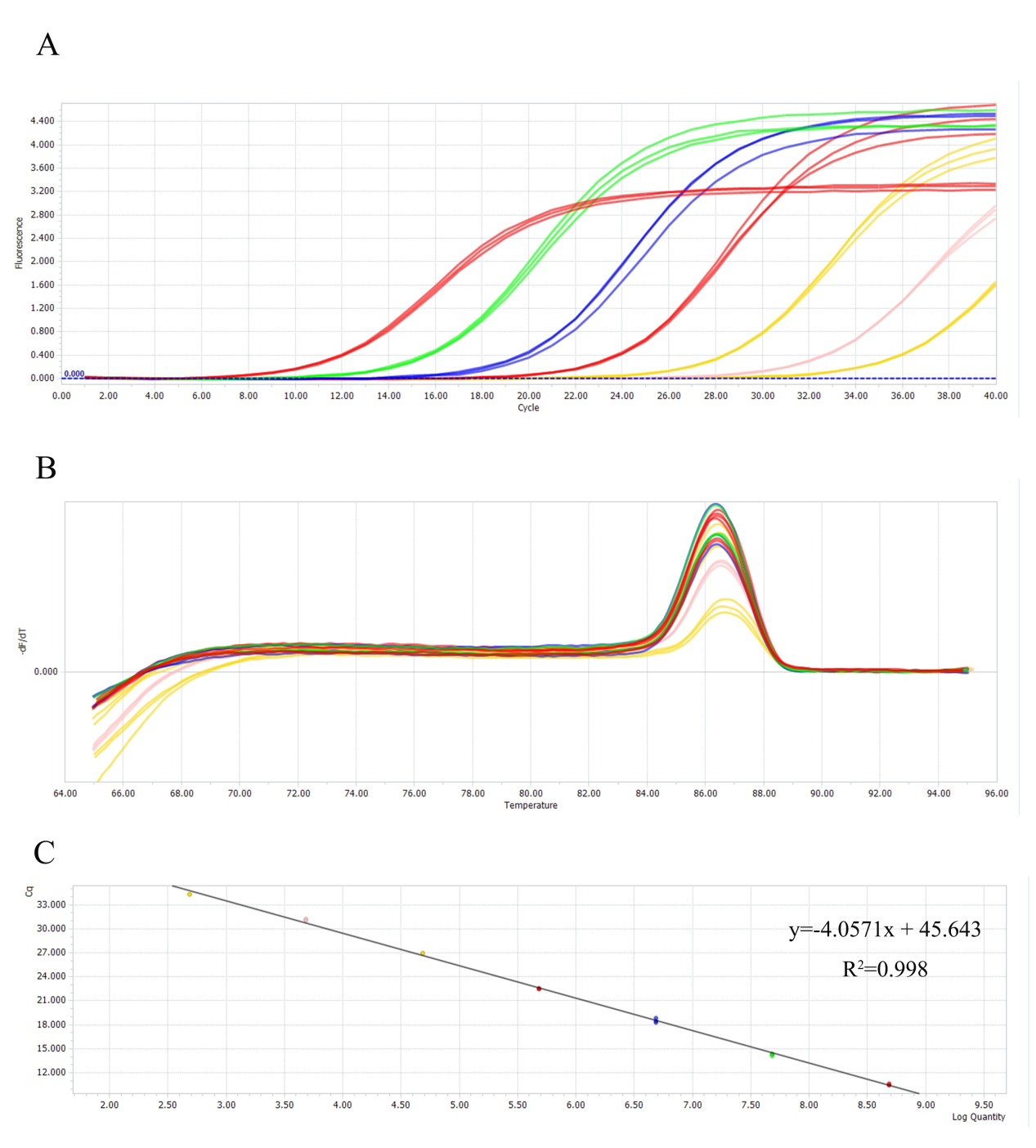


**Fig.S2 Establishment of the *pld* gene fluorescence quantitative RT-PCR detection method**. **(A)** Amplification curves of PGM-T vector plasmid containing gene *pld* with serial dilutions ranging from 4.86 × 10^2^ to 4.86 × 10^8^ copies/reaction. **(B)** Melt curves detection. A single specific peak appears at Tm=(86.5 ± 0.5)℃. **(C)** The standard curve of the experiment. The Cq values (cycle number) plotted against the initial template PGM-T vector plasmid concentration.


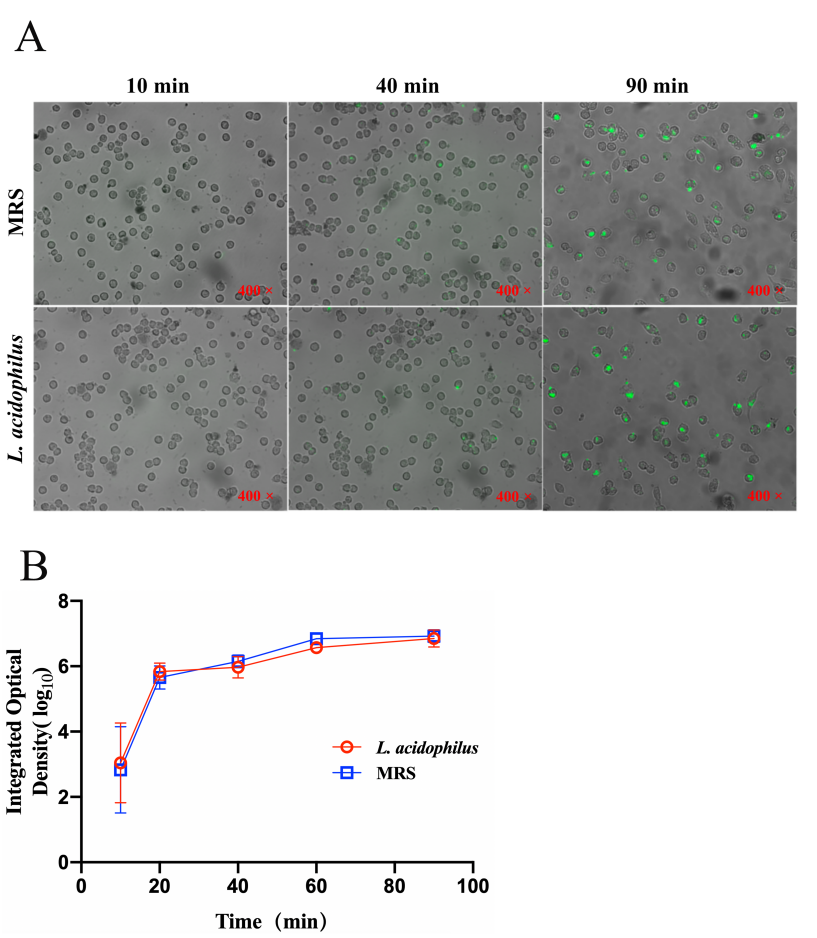


**Fig.S3 Detection of phagocytic capacity of PMs against eGFP-*Escherichia coli***. **(A)** The phagocytic capacity of MPs was assessed against eGFP-*Escherichia coli* through intracellular immunofluorescence counting at different points in time. **(B)** And the statistical results of phagocytic capacity of MPs were expressed by the value of integrated optical density.


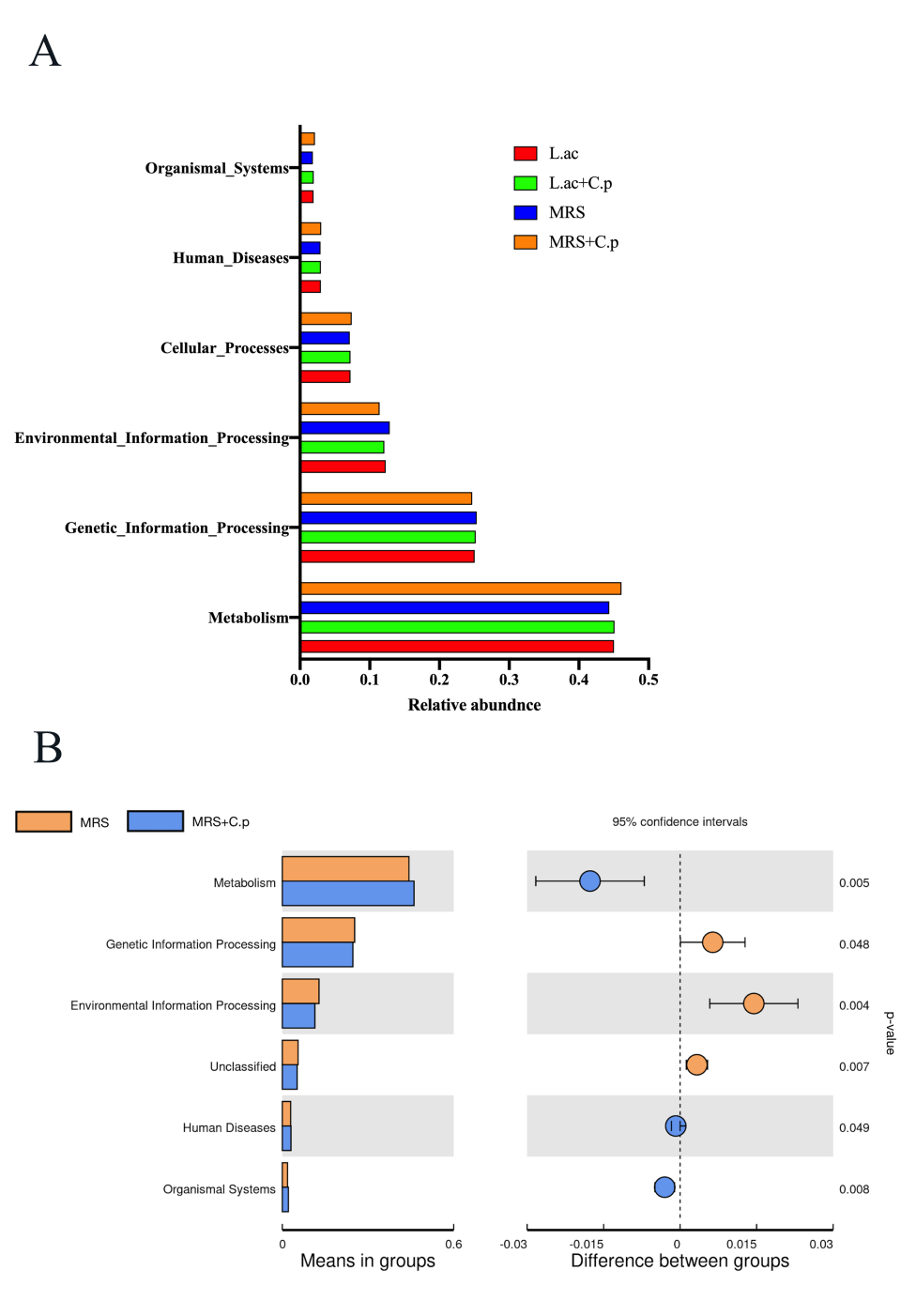


**Fig.S4 Functional analysis prediction**. The Tax4Fun analysis based on the KEGG database to predict microbial metabolic function and analyze the functional differences. **(A)** Comparison of relative abundance of functional annotation genes at the level 1. **(B)** T-test analysis of functional annotation differences between the MRS group and the MRS+*C. p* group.

**Table 1 Primers used in this study**

| **Gene** | **Primer** | **Sequence (5’-3’)** |
| --- | --- | --- |
| *pld* | sense | AGAGTTTGATCCTGGCTCAG |
|  | antisense | GGTTACCTTGTTACGACTT |
| *MCARO* | sense | GACAAGCCCTTCTTCTCGCT |
|  | antisense | AGTTGCTCCTGGCTGGTATG |
| *CLEC7a* | sense | GACTTCAGCACTCAAGACATCC |
|  | antisense | TTGTGTCGCCAAAATGCTAGG |
| *IFN-γ* | sense | ATCTGGAGGAACTGGCAAAA |
|  | antisense | TGAGCTCATTGAATGCTTGG |
| *TNF-α* | sense | CCCTCACACTCAGATCATCTTCT |
|  | antisense | GCTACGACGTGGGCTACAG |
| *IL-6* | sense | AGAAGGAGTGGCTAAGGA |
|  | antisense | GAGAACAACATAAGTCAGATAC |
| *IL-10* | sense | AAGGACCAGCTGGACAACAT |
|  | antisense | TCTCACCCAGGGAATTCAAA |
| *TGF-β* | sense | TGACGTCACTGGAGTTGTACGG |
|  | antisense | GGTTCATGTCATGGATGGTGC |
| *Atg5* | sense | AGAAGATGTTAGTGAGATATGG |
|  | antisense | ATGGACAGTGTAGAAGGT |
| *Atg7* | sense | AGCCCACAGATGGAGTAGCAGTTT |
|  | antisense | TCCCATGCCTCCTTTCTGGTTCTT |
| *Atg12* | sense | CCAAGGACTCATTGACTTC |
|  | antisense | GCAAAGGACTGATTCACATA |
| *Atg16* | sense | TGTCTTCAGCCCTGATGGCAGTTA |
|  | antisense | AGCACAGCTTTGCATCCTTTGTCC |
| *GAPDH* | sense | AGGTCGGTGTGAACGGATTTG |
|  | antisense | TGTAGACCATGTAGTTGAGGTCA |
